# Seeing the heart in 3‐D: Mapping arrhythmias across modalities

**DOI:** 10.1113/JP289664

**Published:** 2025-08-22

**Authors:** Callum M. Zgierski‐Johnston, Jan Huisken

**Affiliations:** ^1^ Institute for Experimental Cardiovascular Medicine, University Heart Center Freiburg ‒ Bad Krozingen Medical Faculty and Medical Center – University of Freiburg Freiburg im Breisgau Germany; ^2^ Multiscale Biology, Department of Biology and Psychology University of Göttingen Göttingen Germany; ^3^ Cluster of Excellence ‘Multiscale Bioimaging: from Molecular Machines to Networks of Excitable Cells’ (MBExC) University of Göttingen Göttingen Germany; ^4^ Morgridge Institute for Research Madison WI USA

**Keywords:** arrhythmia, cardiac electrophysiology, multimodal

Cardiac arrhythmias remain a leading cause of death worldwide. A better understanding of the mechanisms underlying the initiation and maintenance of arrhythmias is necessary for improving clinical interventions. A persistent challenge has been integrating high‐resolution, cellular‐level data with signals relevant to clinical practice. The recent work by Siles et al. (2026) introduces a comprehensive platform that unites panoramic optical mapping, epicardial multi‐electrode arrays and non‐contact torso‐tank mapping in Langendorff‐perfused rabbit hearts, providing a unique opportunity to bridge this translational gap.

Optical mapping remains the gold standard in basic cardiac electrophysiology research to study whole‐heart electrophysiology. However, its conventional application yields only two‐dimensional (2‐D) projections of the inherently three‐dimensional (3‐D) cardiac surface, limiting the ability to fully interpret complex structural‐functional relationships (O'Shea et al., 2020).

Giardini et al. (2025) have advanced the field by employing mathematical techniques to align these 2‐D optical mapping projections with 3‐D structural data obtained by light‐sheet microscopy. Their approach involved generating 2‐D projections of the 3‐D structural data from multiple angles and quantitatively comparing the outlines of these projections to those obtained by optical mapping. The two datasets could thus be aligned by choosing the projection with the minimal difference between outlines. This approach has provided novel insights into how fibrotic regions affect tissue conduction and may cause arrhythmias, highlighting the importance of precise multimodal integration.

Simpler alignment methods are feasible when the modalities used generate datasets that are more directly comparable. de Silva et al. (2024) combined magnetic resonance imaging (MRI) and catheter‐based electro‐anatomical mapping (EAM) *in vivo* in sheep, followed by 3‐D histological analysis of the heart. Despite the significant differences between the methods (EAM generates a 3‐D shell, whereas MRI and histology yield volumetric data), all three generate 3‐D data sets. The shared 3‐D nature of the datasets allows for alignment using shape‐based algorithms and intrinsic anatomical landmarks. This integrative approach enabled the validation of MRI‐based diagnostic techniques and assessment of therapeutic outcomes, paving the way towards improved clinical practice.

Studying a single aspect of physiology (e.g. electrophysiology) across multiple scales and methods is increasingly recognised as essential for advancing mechanistic understanding and informing clinical applications. In this issue of the *The Journal of Physiology*, Siles et al. (2026) present a technique that combines multi‐electrode array measurements, panoramic optical mapping of whole hearts and electrocardiographic imaging (ECGi). ECGi is a non‐invasive technique, where cardiac electrophysiology is computationally reconstructed from body surface electrograms (Kadambadi et al., 2026). Here, the isolated perfused heart is placed within a torso tank model, with electrodes placed on the tank's surface.

By using a motor to rotate the heart during imaging, along with multiple cameras for panoramic optical mapping and advanced mesh refinement, the Siles et al. (2026) generated a high‐resolution 3‐D surface model of the heart. Careful camera calibration enabled precise positioning of the heart model within the torso tank for ECGi, and direct visualisation of the multi‐electrode array allowed for accurate mapping of electrode locations. This integrated approach enabled the combination of ECGi‐based predictions of whole‐heart electrophysiology with direct measurements from both optical mapping and electrodes, all referenced to a single anatomical model.

The resulting multimodal data set, which combines clinically relevant techniques with gold‐standard basic science tools, offers powerful insights into the strengths and limitations of different recording techniques. Such comprehensive integration can improve the identification of arrhythmogenic substrates and, potentially, facilitate the early detection of electrophysiological abnormalities, which may subsequently lead to arrhythmias, using clinically accessible tools.

The studies underscore that combining modalities is not only feasible, but also provides substantially greater insight than single‐modality approaches. However, a key challenge remains: ensuring that these advanced techniques are practical for routine scientific application, as the complexity of the required post‐processing often limits the ability to perform larger‐scale investigations. Future progress will depend on streamlining and automating workflows, as well as extending integration into cellular and even subcellular domains, potentially through integration with omics‐based approaches.

Looking ahead, the integration of multimodal mapping approaches holds immense promise for the future of both research and clinical cardiology. By enabling the direct comparison and validation of experimental and clinically relevant data streams, platforms such as the one developed by Siles et al. (2026) can accelerate the translation of mechanistic discoveries into patient‐specific diagnostics and therapies. As computational modelling and machine learning become increasingly intertwined with physiological research, the wealth of high‐resolution, anatomically registered data generated by these multimodal systems will be invaluable for developing predictive models. The open and modular nature of such platforms encourages adaptation and innovation, empowering others to tailor the methodology to address specific scientific questions.

Ultimately, combining modalities offers a new understanding of both physiological and pathological mechanisms and paves the way for clinical translation. It also serves as a vital tool for validating novel strategies. The methodology described in Siles et al. (2026) stands out as a highly relevant framework for testing and validating new approaches to identify drivers of arrhythmias, with the potential to advance clinical practice and improve patient outcomes.

## Additional information

### Competing interests

No competing interests declared.

### Author contributions

C.Z.‐J. and J.H. were responsible for the conception or design of the work; drafting the work or revising it critically for important intellectual content; and approval of the final version submitted for publication. Both authors agreem to be accountable for all aspects of the work.

### Funding

CZ‐J acknowledges funding from the Deutsche Forschungsgemeinschaft (DFG, German Research Foundation) – 423056183 and 502822458. JH acknowledges funding by the Alexander von Humboldt Foundation (AvH professorship) and the support by the German Research Foundation via the Cluster of Excellence Multiscale Bioimaging (MBExC; EXC 2067/1‐390729940).

## Supporting information


Peer Review History
