# Supplementary material for: Seeing the heart in 3‐D: Mapping arrhythmias across modalities
Source: J Physiol. 2025 Aug 22;604(13):5078–9. doi: 10.1113/JP289664 (PMC13327750; doi:10.1113/JP289664)
Supplement: Supplementary file 1 — Peer Review History [file TJP-604-5078-s001.pdf]

The Journal of Physiology

Seeing the Heart in 3D: Mapping Arrhythmias Across Modalities

Callum Michael Zgierski-Johnston and Jan Huisken
DOI: 10.1113/JP289664

Corresponding author(s): Callum Zgierski-Johnston (callum.michael.johnston@uniklinik-freiburg.de)

The referees have opted to remain anonymous.

Review Timeline:

Submission Date:	16-Jul-2025
Accepted:	29-Jul-2025

Senior Editor: Bjorn Knollmann

Reviewing Editor: Axel Loewe

Transaction Report:

(Note: With the exception of the correction of typographical or spelling errors that could be a source of ambiguity, letters and reports are not edited. Depending on transfer agreements, referee reports obtained elsewhere may or may not be included in this compilation. Referee reports are anonymous unless the Referee chooses to sign their reports.)

1st Editorial Decision 29-Jul-2025

Dear Dr Zgierski-Johnston,

Re: JP-P-2025-289664 "Seeing the Heart in 3D: Mapping Arrhythmias Across Modalities" by Callum Michael Zgierski-Johnston and Jan Huisken

We are pleased to tell you that your paper has been accepted for publication in The Journal of Physiology.

Please see comments below for a small correction that can be made at proof stage.

The last Word (or similar) version of the manuscript provided will be used by the Production Editor to prepare your proof. When this is ready you will receive an email containing a link to Wiley's Online Proofing System. The proof should be thoroughly checked and corrected as promptly as possible.

Authors should note that it is too late at this point to offer corrections prior to proofreading. Major corrections at proof stage, such as changes to figures, will be referred to the Editors for approval before they can be incorporated. Only minor changes, such as to style and consistency, should be made at proof stage. Changes that need to be made after proof stage will usually require a formal correction notice.
All queries at proof stage should be sent to: TJP@wiley.com.

Following copyediting and typesetting the accepted version will be published online.

Yours sincerely,

Bjorn Knollmann
Senior Editor
The Journal of Physiology

IMPORTANT POINTS TO NOTE FOLLOWING YOUR PAPER'S ACCEPTANCE:
If you would like to receive our 'Research Roundup', a monthly newsletter highlighting the cutting-edge research published in The Physiological Society's family of journals (The Journal of Physiology, Experimental Physiology, Physiological Reports, The Journal of Nutritional Physiology, and The Journal of Precision Medicine: Health and Disease), please click this link, fill in your name and email address and select 'Research Roundup':
https://www.physoc.org/journals-and-media/membernews

• TRANSPARENT PEER REVIEW POLICY: To improve the transparency of its peer review process, The Journal of Physiology publishes online as supporting information the peer review history of all articles accepted for publication. Readers will have access to decision letters, including Editors' comments and referee reports, for each version of the manuscript as well as any author responses to peer review comments. Referees can decide whether or not they wish to be named on the peer review history document.

• You can help your research get the attention it deserves! Check out Wiley's free Promotion Guide for best-practice recommendations for promoting your work at: www.wileyauthors.com/eeo/guide. You can learn more about Wiley Editing Services which offers professional video, design, and writing services to create shareable video abstracts, infographics, conference posters, lay summaries, and research news stories for your research at: www.wileyauthors.com/eeo/promotion.

• IMPORTANT NOTICE ABOUT OPEN ACCESS: To assist authors whose funding agencies mandate public access to published research findings sooner than 12 months after publication, The Journal of Physiology allows authors to pay an Open Access (OA) fee to have their papers made freely available immediately on publication.

The Corresponding Author will receive an email from Wiley with details on how to register or log-in to Wiley Authors Services where you will be able to place an order

• You can check if your funder or institution has a Wiley Open Access Account here: https://authorservices.wiley.com/author-resources/Journal-Authors/licensing-and-open-access/open-access/author-compliance-tool.html.

EDITOR COMMENTS

Reviewing Editor:

Thank you for your perspectives article, which nicely contextualizes the study by Siles et al.

Please double check the references as pointed out by the reviewer and implement any corrections during the proofing stage.

Senior Editor:

Thank your for excellent perspective!

REFEREE COMMENTS

Referee #1:

I think the first author of the citated paper is missing. See below the citation extracted from the Journal where it was published.

Leonardo Sacconi, Francesco Giardini, Camilla Olianti et al. Cardiac fibrosis affects electrical conduction and arrhythmogenesis in a pacing-rate-dependent manner, 15 January 2025, PREPRINT (Version 1) available at Research Square [https://doi.org/10.21203/rs.3.rs-5804800/v1
